# The ubiquitin-conjugating enzyme HR6B is required for maintenance of X chromosome silencing in mouse spermatocytes and spermatids

**DOI:** 10.1186/1471-2164-11-367

**Published:** 2010-06-10

**Authors:** Eskeatnaf Mulugeta Achame, Evelyne Wassenaar, Jos W Hoogerbrugge, Esther Sleddens-Linkels, Marja Ooms, Zu-Wen Sun, Wilfred FJ van IJcken, J Anton Grootegoed, Willy M Baarends

**Affiliations:** 1 Department of Reproduction and Development Erasmus MC, Rotterdam, The Netherlands; 2 Department of Biochemistry and Vanderbilt-Ingram Cancer Center, Vanderbilt University School of Medicine, Nashville, USA; 3 Erasmus Center for Biomics, Erasmus MC, Rotterdam, The Netherlands

## Abstract

**Background:**

The ubiquitin-conjugating enzyme HR6B is required for spermatogenesis in mouse. Loss of HR6B results in aberrant histone modification patterns on the trancriptionally silenced X and Y chromosomes (XY body) and on centromeric chromatin in meiotic prophase. We studied the relationship between these chromatin modifications and their effects on global gene expression patterns, in spermatocytes and spermatids.

**Results:**

HR6B is enriched on the XY body and on centromeric regions in pachytene spermatocytes. Global gene expression analyses revealed that spermatid-specific single- and multicopy X-linked genes are prematurely expressed in *Hr6b *knockout spermatocytes. Very few other differences in gene expression were observed in these cells, except for upregulation of major satellite repeat transcription. In contrast, in *Hr6b *knockout spermatids, 7298 genes were differentially expressed; 65% of these genes was downregulated, but we observed a global upregulation of gene transcription from the X chromosome. In wild type spermatids, approximately 20% of the single-copy X-linked genes reach an average expression level that is similar to the average expression from autosomes.

**Conclusions:**

Spermatids maintain an enrichment of repressive chromatin marks on the X chromosome, originating from meiotic prophase, but this does not interfere with transcription of the single-copy X-linked genes that are reactivated or specifically activated in spermatids. HR6B represses major satellite repeat transcription in spermatocytes, and functions in the maintenance of X chromosome silencing in spermatocytes and spermatids. It is discussed that these functions involve modification of chromatin structure, possibly including H2B ubiquitylation.

## Background

The ubiquitin-conjugating enzymes HR6A(UBE2A) and HR6B(UBE2B) are two very similar mammalian homologs of yeast Rad6 [1]. Ubiquitin-conjugating enzymes act together with ubiquitin-activating (E1) and ubiquitin-ligating (E3) enzymes, to conjugate the small 8 kDa ubiquitin protein to specific protein substrates. Poly-ubiquitylation usually targets a substrate for degradation by the proteasome [2], whereas mono-ubiquitylation is involved in various processes including DNA repair and regulation of gene expression [3]. In yeast, Rad6p is involved in different pathways, interacting with different E3 ubiquitin ligase partners. Together with the ubiquitin ligase Ubr1, Rad6 ubiquitylates proteins with destabilizing N-termini [4]. The yeast Rad6-Rad18 heterodimer ubiquitylates proliferating cell nuclear antigen (PCNA) at sites of stalled replication forks during S phase [5]. In addition, Rad6p and the ubiquitin ligase Bre1 are required for ubiquitylation of histone H2B [6-8], a histone modification that is generally associated with ongoing gene transcription [9-11]. Sequential H2B ubiquitylation and deubiquitylation are associated with transcription elongation of certain genes [12,13]. However, Rad6p, and H2B ubiquitylation are also required for silencing of telomeres [14]. Ubiquitylation of H2B by Rad6 depends on phosphorylation of S120 by the cyclin dependent kinases Bur1 and Bur2 [15]. S120A *rad6 *mutants also showed a slow growth phenotype [15]. In human cells, HR6A has also been shown to be a phosphoprotein, and phosphorylation of the conserved S120 residue of HR6A greatly enhanced its in vitro ubiquitylation activity [16].

Chromatin structure depends to a great extent on posttranslational modifications of histones. The combinatorial presence of different acetylations, methylations, phosphorylations, ubiquitylations and sumoylations of the different core histones constitutes the so-called histone code [17], which provides a specific binding platform for regulatory proteins that may also mediate the addition or removal of such posttranslational modifications, or otherwise affect chromatin accessibility.

Ubiquitylation of H2B was shown to be required for subsequent trimethylation of H3K4 and H3K79 [14,18-20]. However, recent data suggest that ubiquitylation of H2B may not be the sole determinant for these modifications in yeast, and that another Bre1 substrate may also be involved [21]. These modifications are known to localize to active chromatin. In mammalian cells, two Bre1 homologs have been identified; RNF20/BRE1A and RNF40/BRE1B, and both appear to be involved in H2B ubiquitylation [22,23]. It has been reported that UBCH6, and not HR6A or HR6B, is the ubiquitin-conjugating enzyme that acts together with RNF20 and RNF40 to ubiquitylate H2B [23]. However, more recent data have shown that depletion of both HR6A and HR6B from human cells results in strongly reduced H2B ubiquitylation [24]. In addition, both Rad6p homologs interact directly with the BRE1A/BRE1B heterodimer [24].

Since HR6A and HR6B differ at only 8 amino acid positions, it is thought that the proteins perform redundant functions, and single gene mutations therefore yield relatively mild phenotypes in mouse [25,26]. *Hr6a *knockout male mice are normal, and females display maternal factor infertility [26]. In contrast *Hr6b *knockout females are normal, and males are infertile [25]. These opposing phenotypes may be due to the fact that HR6A is expressed at a much higher levels than HR6B in oocytes, whereas the reverse is true for developing spermatids [26,27]. This differential gene expression in male and female developing germ cells is related to the fact that HR6A is encoded by the X chromosome [26], and oocytes have two active X chromosomes [28-30], whereas the X chromosome is inactivated during spermatogenesis [31].

As homologous chromosomes pair and align during meiotic prophase, a protein structure is formed that connects the chromosomal axes of the paired chromosomes. This complex is called the synaptonemal complex (SC) (reviewed in [32]). At the zygotene stage of meiotic prophase, pairing has commenced, and the pairing is completed at the pachytene stage. The largely heterologous X and Y chromosomes pair only in the pseudoautosomal region. During the transition from zygotene to pachytene, the presence of unsynapsed sex chromosome arms triggers silencing of X and Y, leading to XY body formation and so-called meiotic sex chromosome inactivation (MSCI) (reviewed in [33]). During postmeiotic spermatid development, several X chromosomal genes are re-expressed or first expressed in the haploid round spermatids, but the average expression level of X chromosomal genes is still low compared to that of autosomal genes [34]. In addition, spermatids, carrying either an X or a Y chromosome, form a distinct postmeiotic sex chromatin (PMSC) area that is enriched for specific histone modifications such as the heterochromatin marker H3K9me3 [34,35]. Recently, Mueller et al [36] showed that the mouse X chromosome is enriched for multicopy genes that show postmeiotic expression. RNA FISH data suggest that the presence of multiple copies enhances the chance of these genes to produce a transcript in a generally repressive chromatin environment [36].

HR6B and RAD18 are both enriched on the XY body of mouse pachytene spermatocytes [37]. Previously, we have shown that mutational loss of *Hr6b *results in specific defects in chromatin structure regulation, visualized by aberrant SC structure and histone modification patterns [38,39]. Surprisingly, global ubiquitylation of H2A and H2B, measured in extracts isolated from *Hr6b *knockout spermatocytes and spermatids, was not affected [39]. In contrast, we observed an increased amount of H3K4me2 specifically on the XY body in spermatocytes and on the sex chromosomes in round spermatids [39]. In addition, H2AT120 phosphorylation was enhanced, first on the XY body of late pachytene spermatocytes, and somewhat later in meiotic prophase also on autosomal chromatin of diplotene and metaphase I spermatocytes. Finally, H3K9me2 was lost from centromeric chromatin in *Hr6b *knockout late spermatocytes and round spermatids [39]. With quantitative RT-PCR (qRTPCR) analysis for selected autosomal and X chromosomal genes, we found a specific derepression of X-chromosomal gene activity for RNA isolated from mixed germ cells and purified spermatids from *Hr6b *knockout mice [39]. From this, we concluded that HR6B controls different histone modifications in spermatocytes and spermatids, which contributes to the postmeiotic maintenance of X chromosome silencing.

Herein, we have performed a global analysis of gene expression in purified spermatocytes and spermatids from wild type and *Hr6b *knockout mice. This allowed us to link the observed changes in the histone code, caused by loss of HR6B, to changes in gene expression in a more global manner. We found a very limited change in gene expression in *Hr6b *knockout spermatocytes compared to wild type cells. In sharp contrast, *Hr6b *knockout spermatids show numerous changes in gene expression. The present results show that *Hr6b *is an important factor that is required for the maintenance of sex chromosome inactivation in both spermatocytes and spermatids. In addition, our data provide novel information about the capability of wild type spermatids to transcriptionally activate a large number of single-copy X-linked genes.

## Results

### HR6A/B is enriched on the XY body and centromeric chromatin of pachytene spermatocytes

**Figure 1:**
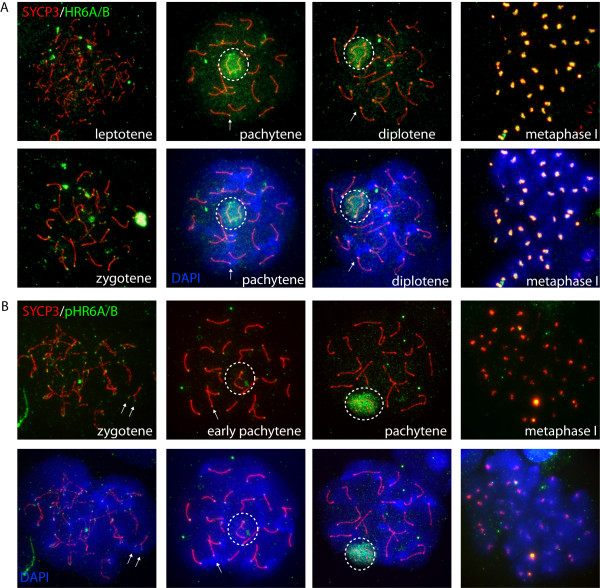
**HR6A/B localizes to centromeric chromatin in early pachytene**. A, B: Immunostaining of spread spermatocyte nuclei for nonphosphorylated HR6A/B (green, A), phosphorylated HR6A/B (green, B), and SYCP3 (red A, B), The merge with DAPI (blue) staining for DNA is shown for a selection of nuclei. The centromeric ends of the SCs can be identified based on the more intense DAPI staining. Arrows indicate centromeric ends that are positive for phosphorylated and non-phosphorylated HR6A/B. The XY body is encircled. During leptotene and zygotene, both phosphorylated and nonphosphorylated HR6A/B accumulate as foci in the nucleus and on the developing SCs. Some centromeric and telomeric ends of the SCs also are enriched for phosphorylated and non-phosphorylated HR6A/B. During early pachytene, the XY body is not enriched for phosphorylated HR6A/B. At this stage, a clear accumulation of phosphorylated HR6A/B is observed on the centromeric ends of the SC. During late pachytene, this staining is lost, and phosphorylated and non-phosphorylated HR6A/B are prominent on the XY body. At metaphase of the first meiotic division, HR6A/B is highly enriched at centromeres, but not phosphorylated.

Previously, we have shown that HR6A/B is enriched on the XY body of pachytene spermatocytes [37]. Using different antibodies that recognize phosphorylated (pHR6A/B) and nonphosphorylated HR6A/B, we performed a more detailed analysis of HR6A/B subnuclear localization throughout meiosis. These antibodies were directed against phosphorylated and nonphosphorylated yeast Rad6 peptides that show high similarity to the analogous sequence of mouse HR6A and HR6B (see Methods). We used an antibody against the synaptonemal complex protein 3 (SYCP3) to substage the meiotic prophase. Figure 1A shows the results of immunostaining for HR6A/B using a polyclonal antibody directed against nonphosphorylated Rad6p. Specific signal was observed as small foci in the nucleus and on the developing SCs in leptotene and zygotene. In pachytene, HR6A/B is enriched on the XY body, and accumulates on the SC ends. During diplotene, HR6A/B is gradually lost from the nucleus, except from the centromeric regions that become highly enriched during metaphase. In spermatids, no specific staining was observed (not shown). Using an antibody against the same phosphorylated epitope (S120), pHR6A/B showed a more clear enrichment on centromeric DNA of early pachytene nuclei, and on the XY body during late pachytene. During later stages, including metaphase, pHR6A/B was absent from centromeric DNA (Figure 1B).

### X-linked spindlin-like gene transcription is upregulated in Hr6b knockout spermatocytes

**Figure 2:**
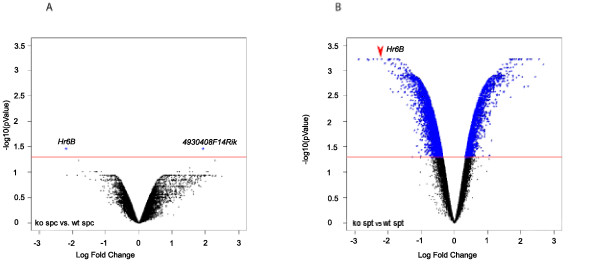
**Volcano plots of p-values (corrected for false discovery) against log2 fold changes**. The log2 fold change is displayed on the x-axis and -log10(p-value), representing the probability that the gene is differentially expressed, on the y-axis. The blue labels indicate genes that are differentially expressed, with a p-value > 0.05. Red lines indicate the p-value cutoff point (0.05; -log10(0.05) = 1.30103). A: Plot showing the p-values derived from the comparison between late spermatocytes from wild type (wt) and *Hr6b *knockout (ko),. Only two differentially expressed genes were found, shown as blue dots. B: Plot showing the p-values derived from the comparison between round spermatids (spt) from wildtype (wt) and *Hr6b *knockout (ko). All 7289 differentially expressed genes are shown as blue dots.

**Figure 3:**
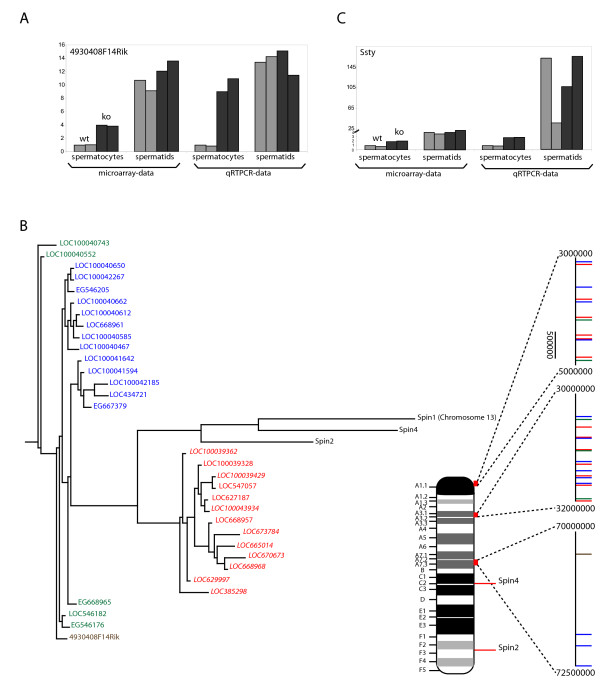
**Premature expression of the X-linked multicopy *spindlin-like *gene in *Hr6b *knockout spermatocytes**. A: Comparision between the array data and qRTPCR data of *4930408F14Rik *mRNA expression in two batches of wild type and *Hr6b *knockout spermatocytes and spermatids. B: Graphic representation of the localization of the multicopy *Spin *gene family on the mouse X chromosome. C: Comparision between the array data and Q-RTPCR data of *Ssty1 *mRNA expression in two batches of wild type and *Hr6b *knockout spermatocytes and spermatids.

RNA isolated from highly purified samples of spermatocytes and spermatids from wild type and *Hr6b *knockout mouse testes was used for microarray analysis. Following data normalization and statistical analyses, we found only 1 gene, apart from *Hr6b*, that was differentially expressed in *Hr6b*^-/- ^spermatocytes. In contrast, 7298 (p-value 0.05, false discovery rate (FDR) corrected) probes were up- or downregulated in *Hr6b*^-/- ^spermatids. At a lower cutoff (p-value 0.01, FDR corrected), we found 2640 probes to be differentially expressed. An overview of the changes in gene expression in spermatocytes and spermatids is given in Figure 2, and the list of differentially expressed genes is available in additional file 1. The single gene that was differentially expressed in spermatocytes, is a protein encoding gene named *4930408F14Rik*. It showed a 3.8 fold upregulation in *Hr6b*^-/- ^spermatocytes on the array, and 10.8 fold upregulation using qRTPCR analysis (Figure 3A). *4930408F14Rik *is an X-linked gene, encoding a 236 aa protein which contains three spindlin/spermiogenesis-specific protein (SPIN-SSTY) domains (IPR003671). The open reading frame encoded by *4930408F14Rik *shows high similarity to the spindlin proteins SPIN1 (56%), SPIN2 (66%) and SPIN4 (54%). *Spin1 *is located on chromosome 13, whereas *Spin2 *and *Spin4 *are on the X chromosome. Further analysis showed that *4930408F14Rik *is a member of a multicopy *spindlin-like *gene family on chromosome X. The X chromosome contains a relatively high number of multicopy genes that are expressed in spermatids. Mueller et al. [36] identified 33 testis-expressed multicopy genes on the X chromosome, and they identified 11 copies of a gene family encoding proteins containing one or more SPIN-SSTY domains, named *EG668965 *and distributed over 2 amplicons. We performed a blast search with the sequence of *4930408F14Rik *and identified a total of 32 closely related family members (excluding *Spin1 *and *Spin4*) on the X, that are localized to three clusters on the X chromosome (Figure 3B). In addition, the Y chromosome encodes the *spindlin-like *genes *Ssty1 *and *Ssty2*, and many other *Ssty*-like (pseudo)genes. Although not statistically significant, the array data revealed a 2.7 and 3.5-fold upregulation for *Ssty1 *(p-val 6.31E-05 before FDR correction, and p-val 0.113 after FDR correction) and *Ssty2 *(p-val 0.000184 before FDR correction and p-val 0.113 after FDR correction) in spermatocytes, but not in spermatids, of *Hr6b *knockout mice. Interestingly, *Spin1 *and the X-encoded *Spin2 *and *Spin4 *were not differentially expressed. We verified the upregulation of *Ssty1 *using qRTPCR (Figure 3C).

### Overall repression of autosomal gene expression in Hr6b knockout spermatids

**Figure 4:**
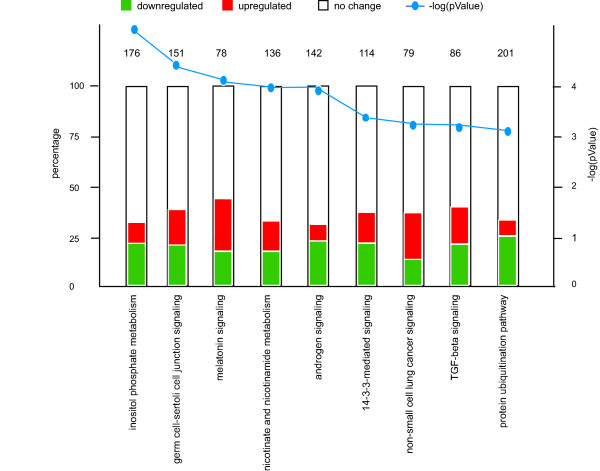
**Pathway analysis**. Differentially expressed genes were uploaded to Ingenuity pathways analysis software. The top 9 pathways are displayed, and the full table is available as additional data file 3. The canonical pathways that are involved in this analysis are displayed along the x-axis. On the y-axis, the percentage of genes that are up- or downregulated is represented. Red shows upregulated, green shows downregulated. The number on the bar graph shows the number of known genes in this pathway, e.g. from the 201 genes that are involved in protein ubiquitination 49 are downregulated and 16 are upregulated; the other 136 genes that are involved in this pathway are not uploaded to Ingenuity because they were not differentially expressed, or for other annotation reasons. The blue line shows the -log (p-value), and the scale shown on the right side of the graph.

In spermatids, approximately 29% of the annotated genes were found to be differentially expressed, and the majority of these genes (66%) were downregulated (Figure 2B). We verified the differential expression of several of the most significantly differentially expressed genes using qRTPCR (additional file 2). These results indicate that HR6B deficiency has an enormous effect on gene expression in round spermatids. To obtain more insight in the regulatory pathways that are affected in *Hr6b *knockout spermatids, 7298 probes that showed significant up- or downregulation were uploaded to Ingenuity pathway analysis software. 6928 probes could be mapped to a gene. From these, 4000 were 'network eligible', meaning that they encode molecules which interact with other molecules present in Ingenuity's knowledge base, and 3472 genes were 'functions eligible', which implies that the encoded proteins have at least one functional annotation or disease association in the knowledge base. In accordance with the fact that quite an abundant number of genes were differentially expressed, many gene networks were found to be affected in *Hr6b *knockout spermatids. The most affected gene networks include post-translational modification, cancer, cell cycle, cell morphology, cellular assembly and organization, RNA post-transcriptional modification, DNA replication, recombination and repair, and embryonic development (additional file 3). When we analysed which canonical pathways are affected by loss of *Hr6b*, the ubiquitin pathway was found in the top 10 (Figure 4). In total, 201 genes of the ubiquitin pathway are included in this database, and 49 of these genes were downregulated and 16 were upregulated in *Hr6b *knockout spermatids; the other 136 genes were not differentially expressed, and therefore not taken into consideration (additional file 4).

### Derepression of major satellite repeat transcription

**Figure 5:**
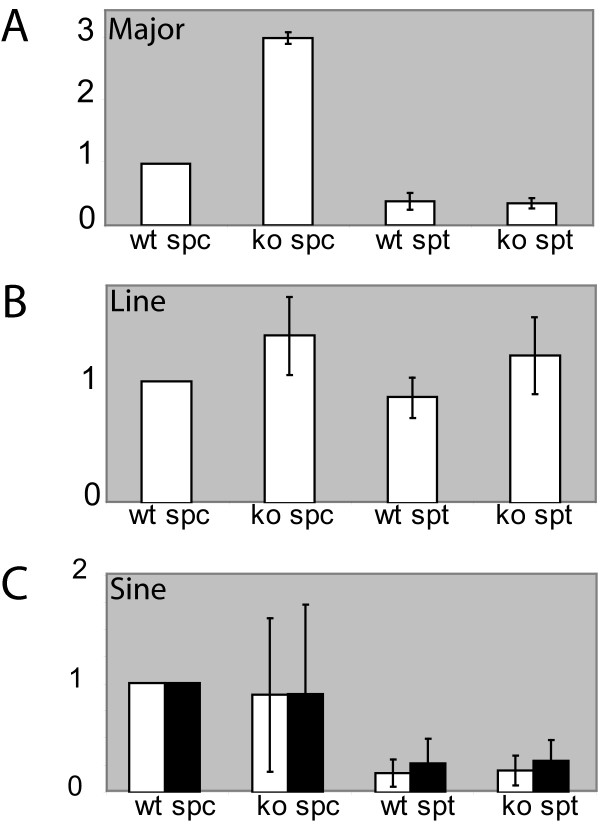
**HR6A/B represses transcription of major satellite repeats in spermatocytes**. Expression of major satellite (A), Line L1 (B), Sine B1 (white bars, C), and Sine B2 (black bars, C) repeats was analysed using Q-RTPCR. The values of all samples were first normalized against β-actin, and subsequently the average value of wild type spermatocytes was set at 1. Error bars indicate the standard deviation.

Previously, we have observed global increases in H2AT120 phosphorylation and macroH2A levels in late spermatocytes of *Hr6b *knockout mice [39], and these changes might lead to the large overall changes in gene expression in spermatids. One of the most striking aberrations observed in *Hr6b *knockout spermatocytes (at diplotene) and spermatids, is the loss of H3K9me2 from centromeric DNA [39]. H3K9me2 and H3K9me3 are known to be enriched at heterochromatic non-genic regions of the genome, including (peri)centromeric DNA regions [40,41], that contain the major and minor satellite repeats. Therefore, we also analysed whether transcription of the major and minor satellite repeats might be affected. To this end, we performed RTPCR and qRTPCR experiments using the same RNA samples that were used for the array. We also analysed transcript levels of several other repeat sequences in the genome (see Methods). Upregulation of major satellite repeat RNA levels was found for *Hr6b *knockout spermatocytes. However, for spermatids, this effect was not observed (Figure 5A). Analysis of other repeat elements in the genome revealed either a very low expression in all samples (Mariner, Charlie, LTR transposons, rDNA, and minor satellite repeats; not shown) or no significant differences between wild type and knockout samples (Line L1, Sine B1, Sine B2; Figure 5B, C).

### Global upregulation of X-linked gene transcription in Hr6b knockout spermatids

**Figure 6:**
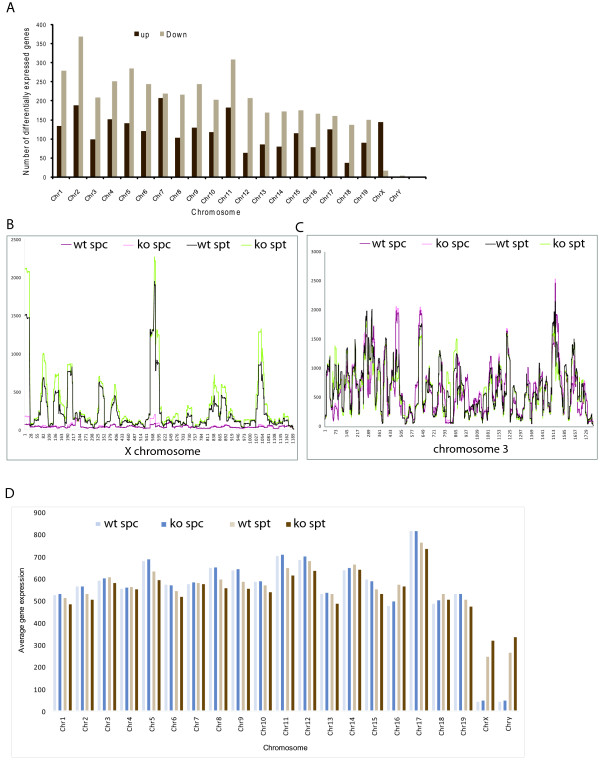
**Global upregulation of X-linked genes in *Hr6b *knockout spermatocytes**. A) Number of differentially expressed genes per chromosome. Significantly expressed genes were annotated and plotted per chromosome. Except for chromosome 7 and chromosome X, the number of downregulated genes in the *Hr6b *knockout is higher than in wild type spermatids. A reverse effect is observed for chromosome X, where the number of upregulated genes in knockout round spermatids is higher than in wild type. Green, downregulated in knockout. Red, upregulated in knockout. B, C) The expression profile along chromosome X (B) and 3 (C) in wild type and *Hr6b *knockout spermatocytes and round spermatids. Gene expression data (linear scale) were mapped to the genomic location of each gene (Affymetrix annotation). D) Average expression per chromosome. RMA normalized average expression for each chromosome was calculated and plotted. Average expression, linear scale, is plotted on the y-axis, and chromosome numbers are shown on the x-axis. The late spermatocytes showed very few and variable changes in average gene expression per chromosome. For round spermatids, the average expression on all chromosomes is slightly higher in wild type, except on chromosome 7 and chromosome X. On chromosome 7 the average expression is equal in wild type and knockout round spermatids. On chromosome X, the average expression is significantly higher in knockout round spermatids. Abbreviations: wt, spt, round spermatid wild type; ko spt, round spermatid *Hr6b *knockout; wt spc, spermatocyte wild type; ko spc, spermatocyte *Hr6b *knockout.

We analysed the chromosomal distribution of all differentially expressed genes to see whether some of these genes might cluster to specific areas. We found that the up- and downregulated genes were randomly distributed, with no apparent clustering to specific chromosomes and regions (not shown). The majority of the genes were downregulated, with the exception of genes on chromosome 7 for which we found an equal distribution of up- and downregulation (Figure 6A, Table 1). In striking contrast, from the 160 genes that were differentially expressed on the X chromosome, 90% were upregulated. Subsequently, we calculated the walking average of gene expression along the X chromosome and along chromosome 3 (that has a length and gene density most similar to X; Table 1) in wild type and *Hr6b *knockout spermatocytes and spermatids. The overall pattern of gene expression along the chromosomes is similar in the two genotypes. However, in this comparison, a global upregulation specifically for the X chromosome in *Hr6b *knockout spermatids was detected (Figure 6B-C). We also compared the average expression level of each chromosome in spermatocytes and spermatids of wild type and *Hr6b *knockout mice (Figure 6D). In spermatocytes, the average expression levels from the autosomes were comparable, and did not differ between wild type and knockout. The average expression level from the X is approximately 10-fold lower due to MSCI, and not different between wild type and knockout. Interestingly, in spermatids, the average expression level is higher in wild type than knockout for all autosomes except for chromosome 17. In contrast, the average X-linked expression is clearly higher in *Hr6b *knockout compared to wild type spermatids.

**Table 1:** Overview of differential gene expression in *Hr6b *knockout spermatids versus wild type per chromosome.

Chrom	Chrom length	# genes	gene density	# differentially expressed	% differentially expressed	up in ko	down in ko	fraction up in ko
1	197195432	1272	155028	412	32.4	134	278	0.33

2	181748087	1942	93588	555	28.6	187	368	0.34

3	159599783	1071	149019	306	28.6	98	208	0.32

4	155630120	1410	110376	402	28.5	151	251	0.38

5	152537259	1339	113919	424	31.7	140	284	0.33

6	149517037	1149	130128	363	31.6	120	243	0.33

7	152524553	2026	75284	426	21.0	207	219	0.49

8	131738871	1121	117519	318	28.4	102	216	0.32

9	124076172	1289	96258	372	28.9	129	243	0.35

10	129993255	1042	124754	319	30.6	117	202	0.37

11	121843856	1748	69704	490	28.0	182	308	0.37

12	121257530	733	165426	270	36.8	63	207	0.23

13	120284312	882	136377	253	28.7	85	168	0.34

14	125194864	879	142429	251	28.6	79	172	0.31

15	103494974	835	123946	289	34.6	115	174	0.40

16	98319150	706	139262	244	34.6	78	166	0.32

17	95272651	1096	86928	285	26.0	125	160	0.44

18	90772031	528	171917	173	32.8	37	136	0.21

19	61342430	758	80927	239	31.5	89	150	0.37

**x**	**166650296**	**965**	**172695**	**160**	**16.6**	**144**	**16**	**0.90**

y	15902555	12	1325213	0	0			

				Total A	Average A	Total A	Total A	Average A

				6391	30.1	2238	4153	0.35

*The first column shows the chromosome (chrom) number, followed by the length in bp, the number (#) of genes, the gene density, the number of differentially expressed genes, the percentage of differentially expressed genes, the number of upregulated genes in *Hr6b *knockout spermatids compared to wild type (up in ko), the number of downregulated genes in *Hr6b *knockout compared to wild type (down in ko), and the fraction of upregulated genes (fraction up in ko). The bottom row shows the total or average values for the columns as indicated for the autosomes only (A).

### X-linked multicopy genes are specifically upregulated from the X chromosome in Hr6b knockout spermatocytes

**Figure 7:**
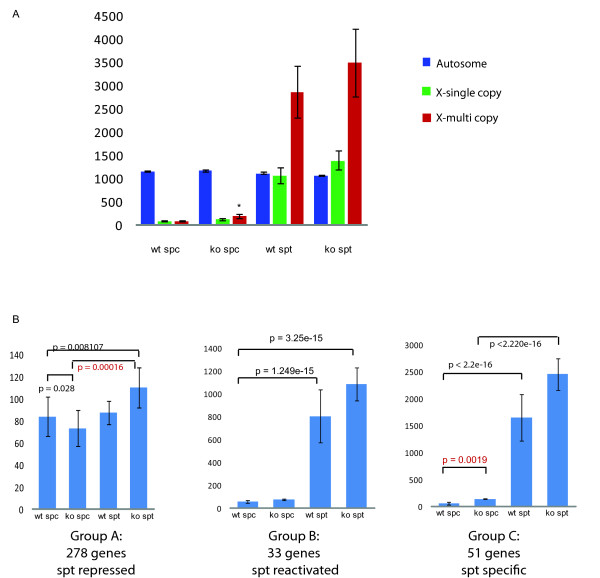
**Analysis of X-linked single-copy and multicopy genes**. A) Average gene expression of autosomal and X-linked multicopy and single-copy genes. In spermatocytes, there is a significant increase in the average expression level of multicopy genes in the knockout compared to wild type. This increase was not observed for single-copy genes. In round spermatids, there is a small increase in the average expression level of both single-copy and multicopy X-linked genes of *Hr6b *knockout compared to wildtype, but this is not statistically significant. The autosomes have a similar average expression in all samples. Genes with an expression value <100 in 3 or more samples were excluded. Asterisk indicates significant compared to wild type (p = 0.05). B) The effect of *Hr6b *knockout on X-linked genes that are repressed in round spermatids (Group A, 278 genes), reactivated in round spermatids (Group B, 33 genes), and genes that are expressed specifically in round spermatids (Group C, 51 genes) as classified by [34]. For group A genes, postmeiotic induction of gene expression is observed only for *Hr6b *knockout spermatids. For group B and C genes a small increase in the average expression was observed in *Hr6b *knockout spermatids, compared to wild type spermatids, but this was not statistically significant. In addition, spermatid-specific X-linked genes show premature expression in spermatocytes of *Hr6b *knockout mice (p = 0.0019)

During postmeiotic spermatid development, most single-copy X- and Y-linked genes remain repressed, but many multicopy and some single-copy genes are expressed from the X and Y chromosomes to high levels [34,36,42]. In the present study, the only gene that showed significant upregulation in *Hr6b *knockout spermatocytes is the multicopy spindle-like gene *4930408F14Rik*. However, in *Hr6b *knockout round spermatids, we observed a global increase in X-linked gene expression, all along the X chromosome. To study whether HR6B might have a differential effect on multicopy versus single-copy X-linked genes, we decided to analyse the expression of the whole group of multicopy genes described by Mueller et al. [36] in more detail, and to compare it to the expression of single-copy genes. In *Hr6b *knockout spermatocytes, compared to wild type spermatocytes, we observed a significant increase in the average expression level of X-linked multicopy genes, but not of single-copy genes (Wilcoxon rank sum test, p-value = 0.0203) (Figure 7A). For the knockout round spermatids, also compared to wild type, the results indicate that there might be a small increase (not statistically significant; p-value = 0.654 for multicopy comparison and 0.262 for single-copy comparison, wilcoxon rank sum test) in the average expression level of both the single-copy and multicopy X-linked genes (Figure 7A). Surprisingly, we found that the average expression level of X chromosomal single-copy genes in spermatids, of both knockout and wild type, was comparable to the average expression level from autosomes. In the data set presented in Figure 7A, all genes that have an expression level of less than 100 in all 4 of the spermatocyte and spermatid fractions were removed. We also observed a marked recovery of X-linked gene expression in spermatids when these criteria were used to calculate the average expression per chromosome (compare Figure 6D to additional file 5, figure A). This approach reveals that 36% of the single-copy genes (199 genes out of 552 X-linked single copy genes selected by Mueller et al. [36]) are re-expressed or first expressed in round spermatids, and can reach levels of transcription that are comparable to what is observed on average for genes expressed from autosomes. Hence, the relatively low average X-linked gene expression level in round spermatids that is observed when all genes are included is mainly due to the fact that many genes have a very low expression level. From the dataset described by Namekawa et al. [34], that was also analysed by Mueller at al. [36], 278 X-linked genes show expression above the arbitrary threshold of 100 in at least 2 out of the four spermatocyte and spermatid samples. When we compared the average expression of this set of genes with the average expression from autosomes (also >100 in at least 2 samples) in spermatids, we observed that the average expression levels of X-linked single-copy and autosomal genes in spermatids were almost similar (additional file 5, figure B), in accordance with the analysis of our own microarray data.

### Single-copy X-linked genes that are spermatid-specific in wild type testis, are prematurely expressed in Hr6b knockout spermatocytes

To further investigate the effect of loss of HR6B on gene expression from the X chromosome, we analysed the X-linked genes as divided in subgroups by Namekawa et al. [34]. They classified X-linked genes based on their expression at different points during spermatogenesis. Of these, 278 genes (including 6 multicopy genes), are repressed at pachytene and remain low in spermatids (Group A). A small subgroup of 33 pachytene-repressed genes (including 1 multicopy gene) are reactivated in round spermatids (Group B), whereas 51 genes (including 8 multicopy genes) are specifically expressed in spermatids (Group C) [34]. We calculated the average expression levels of genes from these groups and compared the levels in wild type and *Hr6b *knockout germ cell fractions. In accordance with the data described by Namekawa et al [34], we observed a low average expression level of group A genes in wild type spermatocytes and spermatids (Figure 7B), and a clear increase in expression for group B genes in spermatids compared to spermatocytes (Figure 7B). Notably, the group A genes show postmeiotic induction of gene expression in the knockout, but not in wild type. In addition, the average expression level of group A and B genes showed a small increase in *Hr6b *knockout spermatids compared to wild types (not statistically significant, p-value = 0.235 for group A and 0.655 for group B wilcoxon rank sum test). For the spermatid-specific Group C genes, we observed premature induction of gene expression in *Hr6b *knockout spermatocytes (p-val 0.0019, wilcoxon rank sum test) (Figure 7B). In addition, the average expression level of these genes was increased in knockout spermatids compared to wild type spermatids, although not statistically significant (p-value = 0.1084, wilcoxon rank sum test). The average expression of autosomal spermatid-specific genes is not different between wild type and *Hr6*b knockout spermatocytes and spermatids (not shown).

### Upregulation of X-GFP protein expression in testes from Hr6b knockout mice

**Figure 8:**
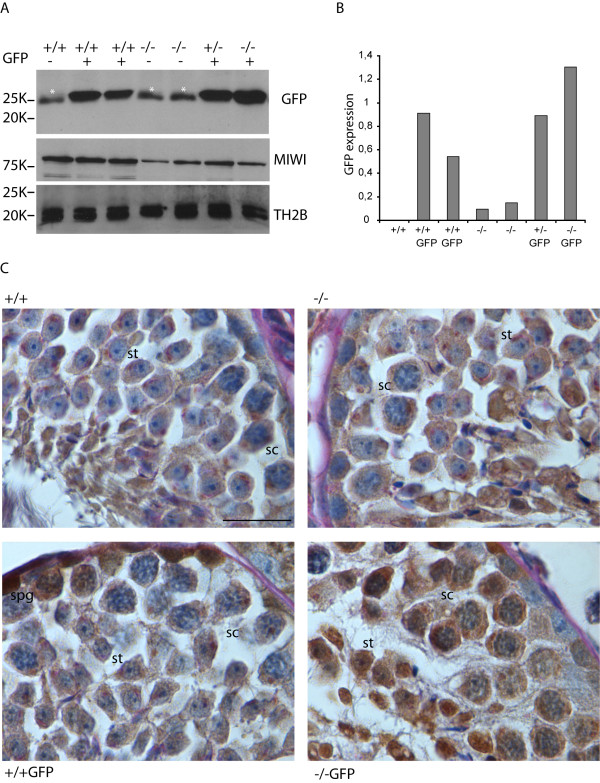
**Enhanced X-linked GFP expression in *Hr6b *knockout testes**. A, Western blot analyses of X-GFP, MIWI and TH2B expression in protein extracts derived from wild type (+/+) *Hr6b*^+/- ^(+/-) and *Hr6b*^-/- ^(-/-) mouse testes with or without the X-linked GFP transgene (GFP). MIWI (expressed in spermatocytes and early round spermatids) and TH2B (expressed from the spermatogonia stage onwards) are shown as controls. The white asterisks indicate a nonspecific band migrating slightly faster than the GFP band. B, quantification of the Western blot data shown in A. GFP signal was quantified using Image J software. The intensity of the background band in lane 1 was subtracted from the GFP signal in all lanes. Subsequently, the signal was normalized to the TH2B signal, and is shown on the Y-axis of the graph. C, immunohistochemical analyses of GFP expression in testis from mice that are wild type (+/+), wild type carrying X-GFP (+/+GFP), *Hr6b *knockout (-/-), or *Hr6b *knockout carrying X-GFP (-/-GFP). Al low level of nonspecific background brown staining is observed in the +/+ and -/- testes. Specific brown GFP staining is observed in spermatogonia (spg) in wild type mice carrying the X-GFP gene, but also in pachytene spermatocytes (spc) and round spermatids (spt) on the *Hr6b *knockout background. Size bar indicates 20 μm.

We also analysed the upregulation of X chromosome gene transcription through a different approach, by asking if the expression of an X-linked transgene would also be dysregulated on the *Hr6b *knockout background. For this, we made use of a mouse line that contains multiple copies of the *GFP *gene at a random location on the X chromosome [43]. Using an antibody against GFP, we detected the protein on Western blots (Figure 8A) and testis sections (Figure 8B) from wild type and *Hr6*b knockout mice that both carried the *GFP *gene on the X chromosome. The level of *GFP *expression, normalized to the level of TH2B, was clearly increased in a total testis extract from approximately 50-day-old *Hr6b *knockout mice compared to age-matched heterozygote and wild type mice (Figure 8A, B). Immunohistochemical analyses showed some background staining in GFP-negative *Hr6b*^+/+ ^and *Hr6b*^-/- ^mouse testis, but the X-linked *GFP *gene is clearly specifically expressed only in spermatogonia of wild type testis, and not during later stages of spermatogenesis. However, on the *Hr6b *knockout background, GFP was also detected in spermatocytes and spermatids (Figure 8C).

### HR6B is not required for H2A ubiquitylation together with UBR2 at the XY body

Very recently, An et al. [44] reported that the E3 ubiquitin ligase UBR2, that complexes with HR6A and HR6B, is required for H2AK119 ubiquitylation in mouse testis [44]. In addition, they observed specific upregulation of X- and Y-linked genes in *Ubr2 *knockout testes, and provided immunofluorescent data that indicate that MSCI does not occur. In contrast, Lu et al. [45] showed, even more recently, that the ubiquitin ligase RNF8 is required for H2A and H2B ubiquitylation during spermatogenesis, and lack of H2AK119 ubiquitylation on the XY body did not interfere with MSCI in their mouse model. In *Hr6b *knockout spermatocytes, we have shown that the level and localization pattern of ubiquitylated H2AK119 are normal, and the initiation of MSCI also occurs normally in *Hr6b *knockout germ cells [39]. In addition, aberrations in XY body chromatin structure of *Hr6b *knockout spermatocytes start to become apparent at the developmental stage when H2Ak119 ubiquitylation is already almost lost from the XY body in wild type spermatocytes. The overall level of H2A ubiquitylation drastically increases as pachytene progresses in both wild type and *Hr6b *knockout spermatocytes ([39] and additional file 6, figure A). In the *Ubr2 *knockout, the UBR2-dependent functions of both HR6A and HR6B might be impaired, whereas we have investigated the effect of loss of HR6B. However, in view of the fact that expression of the X-linked *Hr6a *gene is repressed in spermatocytes [42], and our observations on the defective maintenance of MSCI in *Hr6b *knockout spermatocytes and spermatids, we anticipated a discrepancy between our results and those reported by An et al. [44]. In addition, their data do not match the recent findings of Lu et al [45]. To study this, we analysed the publicly available microarray gene expression data of wild type and *Ubr2 *knockout testes, isolated at postnatal day 16 (when the first wave of spermatogenesis has yielded spermatocytes). We found that, compared to the number of upregulated X- and Y-chromosomal genes, many more autosomal genes show large differences in gene expression level between wild type and *Ubr2 *knockout testis samples (additional file 6, figure B). In particular, we observed that spermatogenesis-specific autosomal genes that are normally first expressed in pachytene [46] show a consistent reduced expression (on average approximately 5-fold) in the knockout sample (Table 2). This is in accordance with an arrest of spermatogenesis in *Ubr2 *knockout mice in early pachytene [44], so that pachytene spermatocytes (in which MSCI occurs) are relatively depleted from the knockout sample. The difference in germ cell composition of the wild type and *Ubr2 *knockout testes provides an alternative explanation for the observed upregulation of X- and Y-linked gene expression; the cells that have reached the stage in which MSCI occurs are present in much lower number in the knockout testes. Also, H2Ak119 ubiquitylation in *Ubr2 *knockout spermatocytes may seem impaired due to the lack of mid- and late-pachytene cells in which the H2AK119ub1 levels are much higher than during early pachytene (additional file 6, figure A). From the above, we suggest that there is lack of evidence that ubiquitylation of H2A and MSCI are impaired in the *Ubr2 *knockout. We hypothesize that, rather than H2A, a yet unknown ubiquitylation substrate of HR6B is required for the maintenance of MSCI. However, we do not exclude the possibility that this substrate is targeted via complex formation between HR6B and UBR2.

**Table 2:** *Ubr2 *knockout testes show 5-fold reduced expression of pachytene-induced genes compared to wild type.

ID_REF	Gene Symbol	Log 2 day 11	log2 day 18	ratio 11/18	Log2 *Ubr2 *ko	Log 2 wt	ratio ko/wt
92929_at	*Cyct*	5,466	6,981	-1,515	9,848	11,482	-1,634

102949_g_at	*Hemt1*	7,008	10,551	-3,542	11,667	11,986	-0,319

93658_at	*Ptpn20*	3,638	6,142	-2,504	9,403	11,482	-2,079

93140_at	*Tsx*	6,571	8,912	-2,341	11,063	11,162	-0,099

92732_at	*Adam2*	1,868	8,318	-6,450	8,598	11,525	-2,927

101864_at	*Actl7b*	3,379	8,461	-5,083	6,267	8,358	-2,091

99995_at	*Cetn1*	3,573	8,130	-4,557	6,715	10,220	-3,505

98334_at	*Crtam*	0,963	5,053	-4,090	4,426	7,084	-2,657

161398_at	*Dnahc8*	4,075	9,146	-5,072	9,849	11,791	-1,942

160219_r_at	*Meig1*	1,233	6,090	-4,857	10,418	12,178	-1,761

101388_at	*Pgk2*	2,379	6,064	-3,685	3,770	8,484	-4,714

99326_at	*Pla2g2c*	6,712	8,569	-1,857	7,098	8,331	-1,233

101938_at	*Pabpc2*	5,849	9,399	-3,550	6,391	9,510	-3,119

93207_at	*Acr*	3,739	7,684	-3,945	6,097	8,689	-2,592

99542_at	*Pdha2*	7,661	11,078	-3,417	10,434	11,366	-0,932

99531_at	*Syngr4*	5,309	8,498	-3,189	8,658	10,180	-1,522

100358_s_at	*Tcp10a/c*	5,609	9,042	-3,433	5,171	9,343	-4,172

103058_f_at	*Tcp10a/b/c*	1,000	8,203	-7,203	5,689	10,602	-4,912

97381_s_at	*Tcp11*	5,642	9,575	-3,933	7,055	9,709	-2,654

93955_at	*Zpbp*	4,961	7,196	-2,235	7,822	9,488	-1,665

100526_f_at	*Adam3*	2,935	7,698	-4,763	5,518	9,798	-4,280

104238_at	*Art5*	6,036	6,990	-0,954	5,873	6,177	-0,304

92911_at	*Ccna1*	4,612	5,957	-1,345	4,961	7,436	-2,475

92198_s_at	*Daf2*	5,081	6,870	-1,789	4,466	7,113	-2,646

97480_f_at	*Dnajb3*	7,828	10,902	-3,075	6,795	9,034	-2,239

102795_at	*Mesp1*	4,225	6,013	-1,788	5,869	8,299	-2,430

161033_at	*Papolb*	4,717	6,695	-1,978	6,181	9,108	-2,928

102244_at	*Tesp1*	4,217	5,044	-0,827	4,435	7,249	-2,813

			Average	-3,321		Average	-2,380

Expression of pachytene-induced spermatogenesis genes at postnatal day 11 and day 18 of testis development [46], and at postnatal day 16 in wild type and *Ubr2 *knockout testis [44]. The ratios of the Log2 values of gene expression in total testis between day 11 and day 18 of wild type and between *Ubr2 *knockout and wild type testes are shown. The bottom row shows the average Log2 values of the ratios between day 11 and day 18 and between wild type and *Ubr2 *knockout testes.

## Discussion

The ubiquitin-conjugating enzymes HR6A and HR6B perform essential functions, as evidenced by the observation that double knockout embryonic stem cells cannot be obtained [26]. In *Hr6b *knockout testes, progression of spermatogenesis is severely affected, and HR6B most likely performs multiple functions at different steps of spermatogenesis [25]. The aberrant structure of the SC in late spermatocytes and the increased frequency of meiotic recombination in *Hr6b *knockout spermatocytes [38] indicate that HR6B might regulate chromatin structure. Herein, we have analysed HR6A/B localization during male meiotic prophase and performed global gene expression analyses to obtain more insight into the effects of HR6B-deficiency on gene expression, in particular of X- and Y- encoded genes in spermatocytes and spermatids. Despite the clear aberrations in chromatin structure in *Hr6b *knockout spermatocytes, we observed very few significant changes in mRNA expression in these cells. This indicates that the aberrations in SC structure and meiotic recombination are most likely due to loss of direct effects of HR6B at the protein level, without robust changes in gene expression. We have previously suggested that the increased meiotic recombination frequency upon loss of HR6B could be caused by a more open chromatin structure, allowing SPO11 to cut at more sites [38]. If this is so, this open chromatin structure does not lead to a significant global increase in gene expression in *Hr6b *knockout spermatocytes.

### HR6B prevents premature expression of postmeiotic X and Y-linked genes in spermatocytes

The only significantly upregulated gene in spermatocytes is the X-linked s*pindlin-like *multicopy gene. Of the 32 copies of this gene that localize to the X-chromosome, only 14 contain an open reading frame of 236 aa. The other copies encode smaller open reading frames of variable length. The Y-linked copies; the *Ssty *genes, were also found to be more highly expressed in knockout spermatocytes compared to controls, but the effect was less pronounced. It is not known whether the *Spindlin-like *genes are actually male fertility genes and if the spindlin-like proteins have a function. When we grouped all known X-linked multicopy genes and analysed their expression levels on the array, we found that the whole group of X-linked spermatid-specific multicopy genes showed premature expression in spermatocytes. In addition, when we grouped the X-chromosomal single-copy genes based on their expression pattern (postmeiotic repression, A; postmeiotic re-expression, B; postmeiotic-specific, C), we found that also the group of single-copy X-linked genes --that are normally spermatid-specific-- show premature induction of their expression. Based on the observed increased H3K4me2 level on the XY body in late spermatocytes [39], we suggest that this premature induction of X-linked gene transcription occurs in diplotene spermatocytes.

In pachytene and early diplotene spermatocytes, phosphorylated HR6B protein is enriched on the XY body. Phosphorylated HR6B may represent the active fraction of the enzyme, since mutation of the analogous site in Rad6p in yeast (S120) affects cell growth, H2B ubiquitylation and telomeric silencing [15]. In yeast, H2B ubiquitylation facilitates methylation of H3K4 and H3K79 [7,14,21]. H3K4me3 and H3K79me2 are enriched on active chromatin in yeast and mammalian cells [40,47-49]. The overall level of H2B ubiquitylation observed on Western blots was found to be normal in *Hr6b *knockout spermatocytes [39], and the nuclear H3K4me2 and H3K4me3 immunostaining signal was not reduced by loss of HR6B. In fact, H3K4me2 was even increased on XY chromatin from diplotene onwards in *Hr6b *knockout spermatocytes, compared to wild type controls [39]. This indicates that H2B might not be a primary target for HR6B on the XY body. However, ubiquitylated H2B most likely also perform functions independent of H3 methylation [50], including possible direct repression of gene expression [51]. Recently, it has been shown that H2B ubiquitylation stabilizes nucleosomes, and this may directly help to repress transcription at certain sites [52]. Indeed, the repressed proto-oncogenes regulated by RNF20 (the human homolog of yeast Bre1p) display "closed" chromatin structure and yet are highly enriched with both ubiquitinated H2B and H3K4me3 [51]. Based on these observations, H2B ubiquitylation might function to maintain MSCI, in a direct manner, through stabilization of nucleosomal structure, thereby inhibiting access of transcriptional activators to the DNA.

### Phosphorylated HR6B functions in the regulation of major satellite transcription through local effects on chromatin structure

Phosphorylated HR6B is also temporarily enriched on centromeric chromatin of pachytene spermatocytes, and H3K9me2 is decreased in centromeric regions of *Hr6b *knockout diplotene spermatocytes [39]. In accordance with these findings, we have now observed enhanced levels of major satellite repeat transcripts in *Hr6b *knockout spermatocytes. This phenotype is reminiscent of the defective telomeric silencing in *rad6 *mutant yeast cells, which depends on H2B ubiquitylation and downstream H3K4 and H3K79 methylation by the histone methyltransferases Set1p and Dot1p, respectively [14,53-55]. In yeast, the roles of H3K4 and H3K79 methylation in silencing might be indirect, meaning that loss of these modifications leads to redistribution of silencing proteins, to regions that are normally active and enriched for H3K4 and H3K79 methylation, resulting in reduced repression of heterochromatin regions [56]. In an analogous manner, the enhanced transcription from major satellite repeats might be caused by a decrease in H2B ubiquitylation at specific sites, not detected as a measurable change in the overall level of H2B ubiquitylation, and a subsequent redistribution of silencing proteins away from the centromeric regions. However, the localization of HR6B at the centromeric regions in mouse spermatocytes indicates that HR6B might have a direct effect on the local chromatin structure, perhaps also through ubiquitylation of H2B, but at silenced regions, leading to enhanced stability of nucleosomes, and directly helping to maintain repression by preventing access of activation factors to the DNA [52].

### HR6B deficiency has a dramatic effect on overall gene expression in round spermatids

Spermatocytes and spermatids of wild type testis contain about equal levels of HR6B [38]. However, using immunocytochemistry, clear localization of HR6B to the chromatin was observed only in spermatocytes. This indicates that HR6B may localize mainly in the cytoplasm in spermatids, or that it is less tightly attached to the chromatin in postmeiotic cells. The dramatic effect on the overall gene expression pattern indicates that postmeiotic spermatid development is severely dysregulated. We analysed samples of the isolated cell fractions by hematoxilin-eosin staining before RNA isolation to verify the purity of the cells (see Methods), and observed no morphological differences between the wild type and knockout fractions. The large overall effect on gene expression may have several causes. It might be envisioned that defects that already occur in spermatocytes affect gene expression later, in spermatids. Lack of ubiquitylation of critical substrates in spermatocytes and/or spermatids may gradually dysregulate many cellular processes that finally also affect gene expression. Lack of H2B ubiquitylation in spermatids could also be one of the causative factors in the observed phenotype. However, we have not been able to detect any ubiquitylated histone fraction in highly purified round spermatids [57]. This may at least in part be due to de-ubiquitylation occurring during spermatid purification [57]. If reduced H2B ubiquitylation in *Hr6b *knockout round spermatids causes global gene repression, it is to be expected that H3K4 methylation would also be reduced, and this has not been observed [39]. This argues against a critical involvement of HR6B-induced H2B ubiquitylation during round spermatid development, although it is not excluded that H2B ubiquitylation might be affected at certain local sites. Lu et al [45] reported very recently that the ubiquitin ligase RNF8 mediates H2A and H2B ubiquitylation during spermatogenesis. During spermatid elongation, lack of H2A and H2B ubiquitylation in *Rnf8 *knockout spermatids prevents H4 hyperacetylation, and thereby blocks removal of histones [45]. In wild type mouse testis, H4 hyperacetylation precedes the histone-to-protamine transition that is required to compact and protect the genome in the small sperm head [58]. Previously, we have shown that increased H2A ubiquitylation precedes the histone-to-protamine transition, and in *Hr6b *knockout elongating spermatids, H2A ubiquitylation appears normal [57]. In addition, the histones are removed, as evidenced from the fact that transition proteins accumulate in elongating *Hr6b *knockout spermatids [25]. Based on these observations, it appears unlikely that HR6B acts together with RNF8 to ubiquitylated H2A and H2B during spermatid elongation. However, it cannot be excluded that RNF8 also interacts with HR6A, which is postmeiotically induced [42], and this may allow RNF8 to ubiquitylate histones in *Hr6b *knockout mouse testis.

### HR6B deficiency leads to global de-repression of X chromosome transcription in round spermatids

During postmeiotic spermatid development, the sex chromosomes are still marked by the presence of silencing proteins, and a low level of Cot-repeat RNA FISH signal [35]. This indicates that there is an overall repression of gene expression from these chromosomes. Indeed, the average gene expression level from the X chromosome is still much lower compared to autosomes in spermatids [34,35]. However, if genes with expression below 100 are excluded from the analyses, the average expression level of X-linked and autosomal genes in spermatids is similar. This indicates that the X-linked single-copy genes that are expressed, can be expressed at normal levels, despite the fact that the X chromosome is generally enriched for heterochromatin markers. The X-linked multi-copy genes have an average expression level that is even higher (approximately 2.5-fold higher than the autosomal average). In *Hr6b *knockout spermatids, we found a somewhat higher expression for X-linked single and multi-copy genes compared to wild type spermatids, and the walking average of gene expression along the X chromosome is higher all along the chromosome. This indicates that the role of HR6B in repression of postmeiotic sex chromosome-linked gene transcription is general and global, consistent with the observed overall increase in H3K4me2 levels on X and Y chromatin in spermatids that was previously observed [39].

Apart from *Hr6b*, one other gene, named *Sly*, was recently shown to regulate postmeiotic sex chromosome inactivation. This is a multicopy Y-linked gene that is specifically induced during postmeiotic spermatid development. Coquet et al. [59], knocked down *Sly *and found specific derepression of sex chromosome-linked gene transcription. The expression of some autosomal genes was also altered, but the majority of differentially expressed genes was X-linked. HR6B has a more global effect on gene expression, involving up and downregulation of many autosomal genes. In addition, it specifically represses X- and Y-linked gene transcription. The number of X- and Y-linked genes that is differentially expressed in *Sly *knockdown and *Hr6b *knockout spermatids is comparable. Direct comparison of the datasets is difficult because different array platforms were used. However manual comparison revealed that approximately 20-30 X- or Y-linked genes are upregulated in both models. Deficiency for SLY leads to decreased levels of H3K9me3 on X and Y chromatin in spermatids [59]. In *Hr6b *knockout spermatids, such a decrease has not been observed. Instead, we found increased H3K4me2 on X and Y [39]. In addition, HR6A and HR6B are not differentially expressed in the *Sly *knockdown spermatids [59]. Together, these data indicate that HR6B and SLY might act on postmeiotic sex-linked gene transcription via independent mechanisms.

## Conclusions

Although HR6B is required for several aspects of chromatin dynamics through the male meiotic prophase, the overall gene expression is hardly affected in *Hr6b *knockout spermatocytes. Still, single-copy and multicopy X-linked postmeiotic genes are expressed prematurely in *Hr6b *knockout spermatocytes. In addition, HR6B represses major satellite repeat transcription, a function reminiscent of the role of Rad6 in telomeric silencing in yeast.

In *Hr6b *knockout spermatids, 30% of the annotated genes is differentially expressed, and multiple functional pathways are affected to a similar extent, indicating gross overall dysregulation of gene transcription. In wild type spermatids, single-copy postmeiotic X-linked genes reach expression levels that are comparable to that of autosomal genes. This indicates that approximately 20% of gene promoters on the X chromosome can be normally activated, despite the presence of heterochromatin markers originating from MSCI. Previously, RNA polymerase II mediated gene transcription of miRNAs has also been reported in spermatocytes as well as spermatids [60]. We suggest that the overall repressed conformation of the sex chromosomal chromatin, both in spermatocytes and spermatids, does not interfere with access of the transcription machinery to specific genes. This is consistent with the fact the DAPI staining of the XY body and of PMSC is not as dense as for example the DAPI staining of centromeric chromatin.

Lack of HR6B, in association with global upregulation of H3K4me2, leads to a global upregulation of X chromosomal gene expression in spermatids,. The critical ubiquitylation substrate for HR6B at the XY body and PMSC is not known. The E3s UBR2 and RAD18 both interact with HR6A/B, and also accumulate at the XY body. For the Bre1 homologs, RNF20 and RNF40, the localization in meiotic cells is not known. Either one of these E3s, or an unknown E3, may be involved in targeting a chromatin component for ubiquitylation by HR6B to maintain sex chromosome silencing in spermatocytes and spermatids.

## Methods

### Isolation of different cell types from mouse testis

All animal experiments were approved by the animal experiments committee DEC-Consult (EUR 897, EUR1168, EUR 1427). To obtain knockout mice, *Hr6b*^+/- ^mice, obtained from backcrosses of *Hr6b*^+/- ^mice with FVB mice, were intercrossed to obtain *Hr6b*^-/- ^females. These were then crossed with Hr6b^+/- ^males derived from backcrosses of *Hr6b*^+/- ^mice with FVB mice, to yield male *Hr6b*^-/- ^mice. *Hr6b*^+/+ ^mice, derived from backcrosses of *Hr6b*^+/- ^mice with FVB mice were intercrossed to yield male *Hr6b*^+/+ ^mice. The crosses to obtain male *Hr6b*^+/+ ^and male *Hr6b*^-/- ^mice were set up simultaneously, using animals from the same backcross generation (>20).

Spermatocytes and round spermatids were isolated from 4-5-week-old *Hr6b*^+/+ ^and *Hr6b*^-/- ^mouse testes after collagenase and trypsin treatment, followed by sedimentation at unit gravity (StaPut procedure). Subsequently, cells were further purified by density gradient centrifugation through Percoll [61]. Cells were snap-frozen in liquid nitrogen and stored at -80°C. A sample of each isolated fraction was used for hematoxilin-eosin staining on slides, in order asses the purity of the fractions. The purity of the cells was >80%, and not different between wild type and knockout preparations.

### RNA isolation and qRTPCR

For real-time qRTPCR and array hybridization, RNA was prepared from the isolated germ cell preparations by Trizol. The purity, integrity and quality of extracted RNA were measured using Nanodrop and Bioanalyzer 2100 (Agilent technologies). Prior to the reverse transcriptase reaction, RNA was DNAse-treated (1 μl RNase free DNase with 2 μg RNA in a total reaction volume of 15 μl for 30 minutes at 37°C, followed by heat inactivation of the enzyme at 65°C for 10 minutes). Subsequently, the reverse transcriptase reaction was carried out in 40 μl reaction volume containing 0.5 mM dNTPs, 2.5 ng/μl random hexamer primers, 5 mM MgCl2, 0.01 M DTT, 2 μl RNase-out, 2.5 U/μl Superscript II Reverse Transcriptase (Invitrogen, Breda, The Netherlands), and 1× reaction buffer supplied by the manufacturer of the enzyme (Invitrogen). Primers were annealed for 10 minutes at 25°C, followed by 50 minutes of cDNA synthesis at 42°C. The reaction was stopped by incubating the mixture at 70°C for 10 minutes. Subsequently, the cDNA was treated with RNase H for 20 minutes at 37°C. Upon heat inactivation, the cDNA was kept at -20°C. 0.4 μl of the cDNA reaction mixture was used for each real-time PCR experiment. This reaction was carried out in a total volume of 25 μl including 12.5 ul SYBR Green PCR Master Mix (Applied Biosystems) and the appropriate primer mixture in the CFX96 Real-Time System (Bio-Rad). The reaction involved 3 minutes 95°C denaturation, followed by 40 cycles of 30 seconds 95°C, 30 seconds, annealing, 30 seconds extension at 72°C, and a final 5 minute incubation at 72°C. Readouts were performed after each cycle. Primer sequences, exon location, annealing temperatures, and product sizes, are shown in additional file 7. Primers for selected genes were designed using the Primer3 program http://frodo.wi.mit.edu/primer3/. Primer Blast http://www.ncbi.nlm.nih.gov/tools/primer-blast/index.cgi?LINK_LOC=BlastNoResAd was used to test the specificity of the primers *in silico*. Primer sequences for selected repeat sequences were taken from Martens et al. [62]. *β-actin *(primers have been described by Namekawa et al. [34]) was included in each reaction and used to normalize the data. The quality of the primers was tested through analysis of the melting curve, which showed a single peak for all selected genes. For the LTR transposon and the Line L1 repeats, the products were melted at 78°C and 74°C, respectively, before performing each readout. For the major and minor satellite repeat sequences, multiple peaks were observed, as expected. Two independent experiments were performed for each analysed gene and each real-time PCR reaction was performed in duplicate. -RT reactions were negative.

### Immunohistochemistry, protein isolation, and Western blotting

50-day-old wild type FVB mice, transgenic mice carrying the X-linked GFP transgene (*X-GFP*), and *Hr6b*^+/- ^and *Hr6b*^-/- ^mice with or without the *X-GFP *gene were killed and one testis was snap frozen in liquid nitrogen and stored at -80°C. The other testis was fixed in 4% paraformaldehyde in PBS at 4°C overnight followed by dehydration and embedding in paraffin according to standard procedures. Protein extracts were prepared by 10 cycles of 10 seconds sonication in 0.25 M sucrose/1 mM EDTA supplemented with complete protease inhibitor cocktail (Roche). Protein concentrations were determined using Coomassie Plus protein assay reagent (Pierce, Perbio Science, Etten-Leur, Netherlands) as described by the manufacturer.

An amount of 20 μg of protein per sample was separated on 12% SDS-polyacrylamide gels and the separated proteins were transferred to nitrocellulose membranes, using the BioRad miniprotean III system and blot cells (Bio-Rad, Veenendaal, Netherlands). Membranes were stained with Ponceau S (Sigma-Aldrich, Zwijndrecht, Netherlands) according to the supplier's protocol.

GFP was detected using a mouse monoclonal anti-GFP (Roche) at a 1:1000 dilution. Rabbit polyclonal MIWI antibody (Cell Signaling) was used at a 1:1000 dilution. Mouse monoclonal anti-TH2B (Millipore, anti-tyrosine hydroxylase) was used at a 1:1000 dilution. After blocking non-specific sites with 3% w/v non-fat milk in PBS with 0.1% v/v Tween20 (blotto) for 1 hour at room temperature, antibody was added in fresh blotto, and incubation was continued for an additional hour at room temperature. Subsequently, non-bound antibody was removed through several washes using PBS with 0.1% v/v Tween20. Peroxidase-labeled second antibody (Sigma) was diluted in blotto, and incubation was for 1 hour at room temperature. Antigen-antibody complexes were detected by using a chemoluminescence kit (Du Pont/NEN, Bad Homburg, Germany) according to the instructions provided by the manufacturer. Immunohistochemistry for X-GFP (anti-GFP at 1:4000 dilution) was performed as described previously (Roest et al., 1996), except that the slides were counterstained using the period acid Schiff (PAS) staining followed by hematoxilin.

### Meiotic spread nuclei preparations and immunocytochemistry

Spread nuclei preparations of mouse spermatocytes were prepared using a modification of the drying-down technique described by Peters et al. [63]. For immunocytochemistry, frozen slides were defrosted at room temperature and washed with PBS. The slides were blocked with PBS containing 0.5% w/v BSA and 0.5% w/v milk powder, and were double stained with rat polyclonal anti-SYCP3 [39], and mouse monoclonal IgM anti-ubi-H2A (Upstate, Waltham, MA, USA) or rabbit polyclonal antibody directed against the yeast Rad6 protein phosphorylated or nonphosphorylated at S120 (NDPNPAS*PANVE). The corresponding sequence in both HR6A and HR6B is (DEPNPNS*PANSQ). Specificity of the antibody for mouse phosphorylated and nonphosphorylated HR6A/B was determined using the peptides for competition experiments (not shown). For rabbit polyclonal primary antibodies, the secondary antibody was fluorescein isothiocyanate (FITC) (Sigma, St Louis, USA)-labeled goat anti-rabbit IgG. The secondary antibody used for the rat polyclonal anti-SYCP3 was Alexa 594-labeled goat anti-rat IgG. FITC-labeled goat anti-mouse IgM (Sigma) was used as secondary antibody for anti-H2A_K119ub1 _(IgM). Primary antibodies were diluted in 10% w/v BSA in PBS and incubated overnight in a humid chamber. Thereafter, slides were washed in PBS, blocked in 10% v/v normal goat serum (Sigma) in blocking buffer (5% milk powder; w/v in PBS, centrifuged at 13,200 rpm for 10 min), and incubated with secondary antibodies in 10% v/v normal goat serum in blocking buffer at room temperature for 2 hours. Next, the slides were washed in PBS and embedded in Vectashield containing DAPI (4',6'-diamidino-2-phenylindole) (Vector Laboratories, Burlingame CA, USA).

### Microarray data generation

The purity, integrity and quality of extracted RNA was measured using Nanodrop and Bioanalyzer 2100 (Agilent technologies). cRNA was produced from purified RNA and labelled according to the Affymetrix sample preparation protocol. Labelled samples were hybridised to Mouse Genome 430 2.0 Arrays following the Affymetrix hybridisation protocol. After ~16 hours, hybridised arrays were washed and signal intensities were generated by scanning arrays using the Affymetrix 7G GeneChip scanner. For each cell type replicate experiments were performed, 8 arrays in total.

### Microarray data analysis

Normalised signal intensities were generated from Affymetrix CEL files (GEO database http://www.ncbi.nlm.nih.gov/geo, accession number GSE21749 for data generated and deposited by us, and accession number GSE4193 for data generated by Namekawa et al. [34]) using RMA normalisation implemented in R software (Bioconductor project, Limma package) [64]. Linear models and empirical Bayes methods, implemented in R Limma package, were used to asses differential expression [65]. To evaluate the expression pattern of genes across different chromosomes, normalized data were exported to excel and annotated using the corresponding annotation file from the array manufacturer (Affymetrix, Mouse430_2 Annotations, Release 30 (11/15/09)). Array probes with less than 100 signal intensity in more than 50% of samples in the normalized data were excluded from the analysis where indicated. To investigate the effect of *Hr6b *knockout on single-copy and multi-copy X-linked genes, 552 single-copy and 25 multi-copy genes described by Mueller et al. [36], were selected in wild type and *Hr6b *knockout normalised microarray data of spermatocytes and spermatids. Data were summarised and plotted in Excel. Statistical tests (Wilcoxon rank sum test) were performed using R software. To understand the effect of HR6B on X-linked genes that are expressed at different stages of spermatogenesis, groups of genes classified by Namekawa et al. [34] were used. As described by Namekawa et al. [34], 278 genes that that are repressed in round spermatids (Group A), 33 genes that are reactivated in round spermatids (Group B), and 51 genes that show specific expression in round spermatids (Group C), were annotated with wild type and *Hr6b *knockout data and plotted using Excel. Statistical test (Wilcoxon rank sum test) was performed using R software.

To understand the pathways that are affected by HR6B, lists of differentially expressed genes were imported and analysed and using Ingenuity software (Ingenuity systems).

## Abbreviations

(PCNA): proliferating cell nuclear antigen; (SC): synaptonemal complex; (MSCI): meiotic sex chromosome inactivation; (PMSC): postmeiotic sex chromatin; (qRTPCR): quantitative RT-PCR; (pHR6A/B): phosphorylated HR6A/B; (SYCP3): synaptonemal complex protein 3; (SPIN-SSTY): spindlin/spermiogenesis-specific protein domains; (FITC): fluorescein isothiocyanate

## Authors' contributions

EMA participated in the design of the study, carried out the microarray analyses, bioinformatics analyses and drafted the manuscript. EW isolated RNA and protein and performed qRTPCR analyses and immunocytochemical and western blot analyses. JWH purified testicular cell types, isolated RNA and performed qRTPCR analyses. ES-L performed qRTPCR analyses. MO isolated tissues and performed immunohistochemical analyses. ZWS generated and characterized antibodies, WFJvIJ generated microarray data and participated in writing the manuscript, JAG participated in the design of the study and writing of the manuscript. WMB conceived of the study, participated in its design and wrote the manuscript. All authors read and approved the manuscript.

## Supplementary Material

Additional file 1 **Differentially expressed genes between *Hr6b *knockout and wild type germ cells**. Sheet 1: differentially expressed genes between *Hr6b *knockout and wild type spermatocytes. Sheet 2: differentially expressed genes between *Hr6b *knockout and wild type spermatids Click here for file

Additional file 2 **Comparision between the array data and qRTPCR data of selected genes in two batches of wild type and *Hr6b *knockout spermatocytes and spermatids**. A, Comparision between the array data and qRTPCR data of selected autosomal genes differentially expressed in *Hr6b *knockout spermatids. B, Comparision between the array data and qRTPCR data of selected autosomal genes with the highest P-value for differential expression in spermatocytes (not significant). C, Comparision between the array data and qRTPCR data of selected X- and Y-linked genes differentially expressed in *Hr6b *knockout spermatids. P-values are indicated. qRTPCR data were normalized to β-actin. Click here for file

Additional file 3 **Pathways affected by *Hr6b *knockout in spermatids**. Differentially expressed genes between *Hr6b *knockout and wild type spermatids were imported into Ingenuity pathway analysis software, and the significant pathways that are affected in this knockout are presented. Click here for file

Additional file 4 **Genes that are known to be involved in ubiquitin pathway and are affected by *Hr6b *knockout**. Differentially expressed genes between *Hr6b *knockout and wild type spermatids were imported into Ingenuity pathway, and their effect on canonical pathways was investigated. In this table the genes that are involved in the ubiquitin canonical pathway, and affected in *Hr6b *knockout spermatids are presented. Click here for file

Additional file 5 **X-linked genes that are (re)induced in spermatids reach an average expression level that is comparable to the average expression from autosomes**. A) Normalized average expression for each chromosome was calculated and plotted, genes with an expression value of less than 100 in 3 or more samples were excluded from the analysis. Average expression, linear scale, is plotted on the y-axis, and chromosome numbers are shown on the X-axis. The late spermatocytes showed very small and variable changes in average gene expression per chromosome. For round spermatids, the average expression from most autosomes is slightly higher in wild type. On chromosome X, a reverse effect is observed, where the average expression is significantly higher in knockout round spermatids. Abbreviations: wt, spt, round spermatid wild type; ko spt, round spermatid *Hr6b *knockout; wt spc, spermatocyte wild type; ko spc, spermatocyte *Hr6b *knockout. B) Average gene expression of autosomal and X-linked genes in spermatocytes and spermatids analysed in the microarray data set from Namekawa et al [34]. Genes with an expression value <100 in 3 or more samples were excluded. Click here for file

Additional file 6 **Analyses of gene expression in *Ubr2 *knockout testis and H2AK119 ubiquitylation in wild type spermatocytes**. A) Analysis of H2AK119 ubiquitylation (H2AK119ub1, green) and SYCP3 (red) during meiotic prophase in spread wild type mouse spermatocyte nuclei. Each image was obtained using the same microscope and camera settings. B) Genes that were more than two-fold up or downregulated in *Ubr2 *knockout versus wild type samples [44] were calculated per chromosome. Click here for file

Additional file 7 **PCR primers used for qRTPCR**. Click here for file
