# Supplementary figures and images for: The ubiquitin-conjugating enzyme HR6B is required for maintenance of X chromosome silencing in mouse spermatocytes and spermatids
Source: BMC Genomics. 2010 Jun 10;11:367. doi: 10.1186/1471-2164-11-367 (PMC3091626; doi:10.1186/1471-2164-11-367)

A

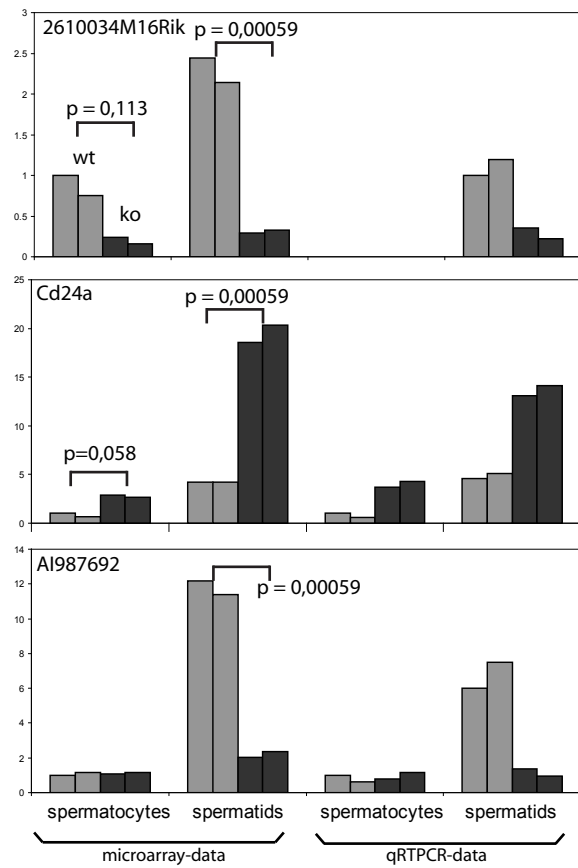

B

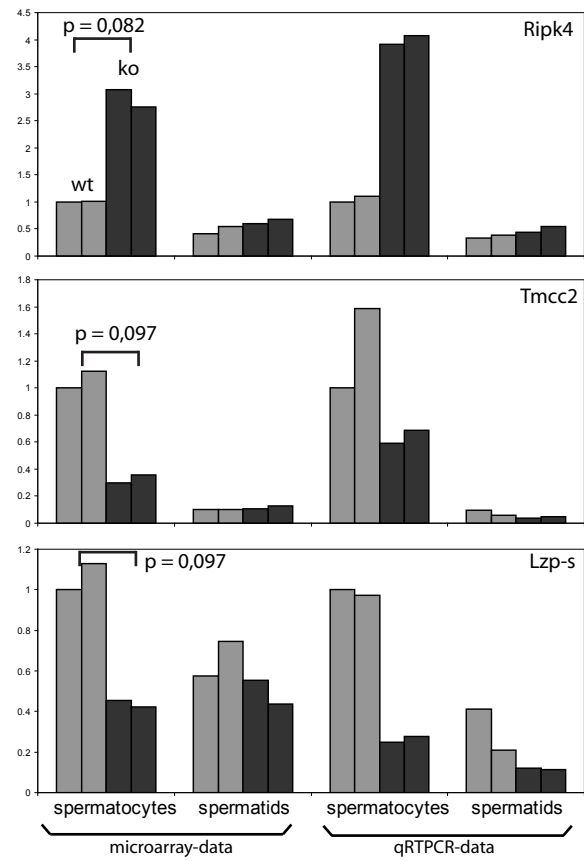

C

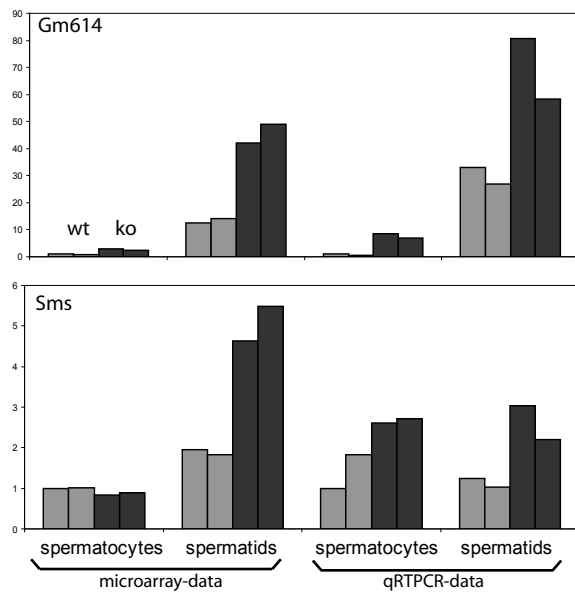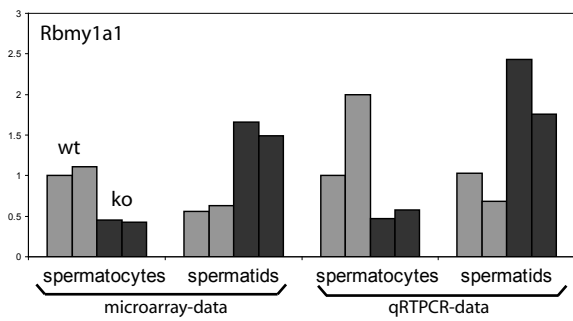

Supplement: Additional file 2 — Comparision between the array data and qRTPCR data of selected genes in two batches of wild type and Hr6b knockout spermatocytes and spermatids. A, Comparision between the array data and qRTPCR data of selected autosomal genes differentially expressed in Hr6b knockout spermatids. B, Comparision between the array data and qRTPCR data of selected autosomal genes with the highest P-value for differential expression in spermatocytes (not significant). C, Comparision between the array data and qRTPCR data of selected X- and Y-linked genes differentially expressed in Hr6b knockout spermatids. P-values are indicated. qRTPCR data were normalized to β-actin. [file 1471-2164-11-367-S2.PDF]

A

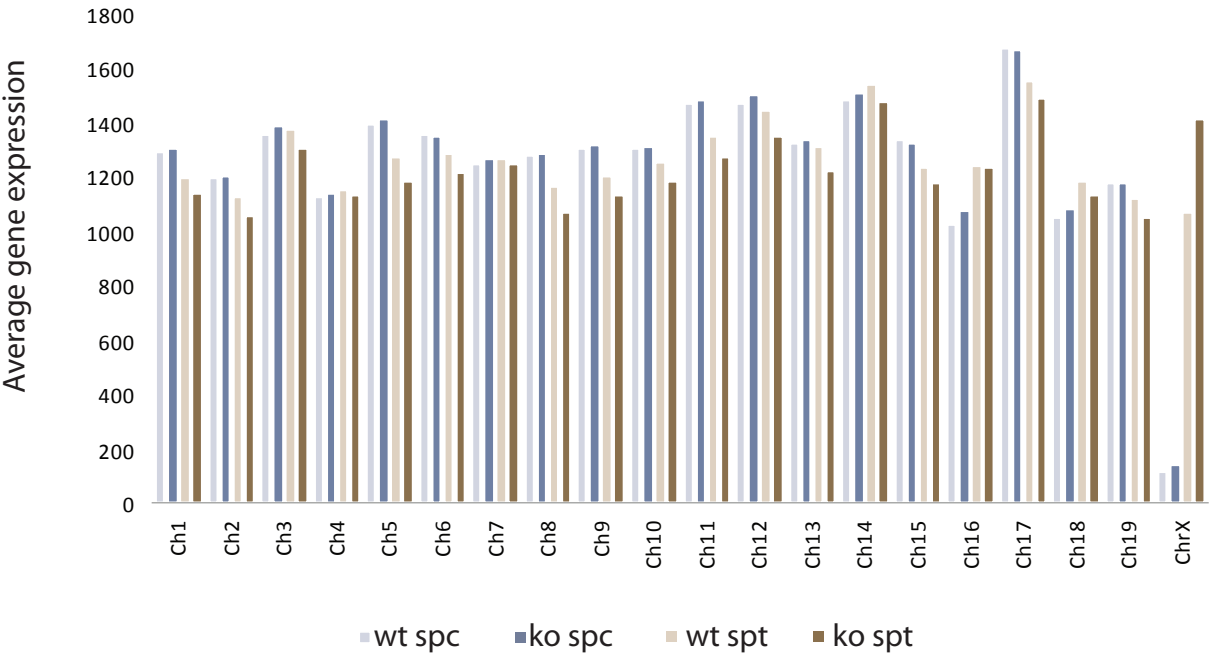

B

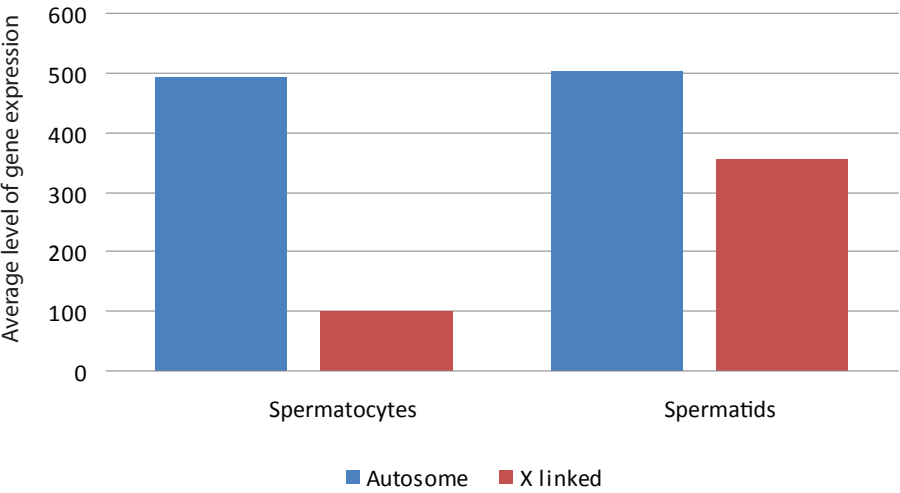

Supplement: Additional file 5 — X-linked genes that are (re)induced in spermatids reach an average expression level that is comparable to the average expression from autosomes. A) Normalized average expression for each chromosome was calculated and plotted, genes with an expression value of less than 100 in 3 or more samples were excluded from the analysis. Average expression, linear scale, is plotted on the y-axis, and chromosome numbers are shown on the X-axis. The late spermatocytes showed very small and variable changes in average gene expression per chromosome. For round spermatids, the average expression from most autosomes is slightly higher in wild type. On chromosome X, a reverse effect is observed, where the average expression is significantly higher in knockout round spermatids. Abbreviations: wt, spt, round spermatid wild type; ko spt, round spermatid Hr6b knockout; wt spc, spermatocyte wild type; ko spc, spermatocyte Hr6b knockout. B) Average gene expression of autosomal and X-linked genes in spermatocytes and spermatids analysed in the microarray data set from Namekawa et al [34]. Genes with an expression value <100 in 3 or more samples were excluded. [file 1471-2164-11-367-S5.PDF]

A

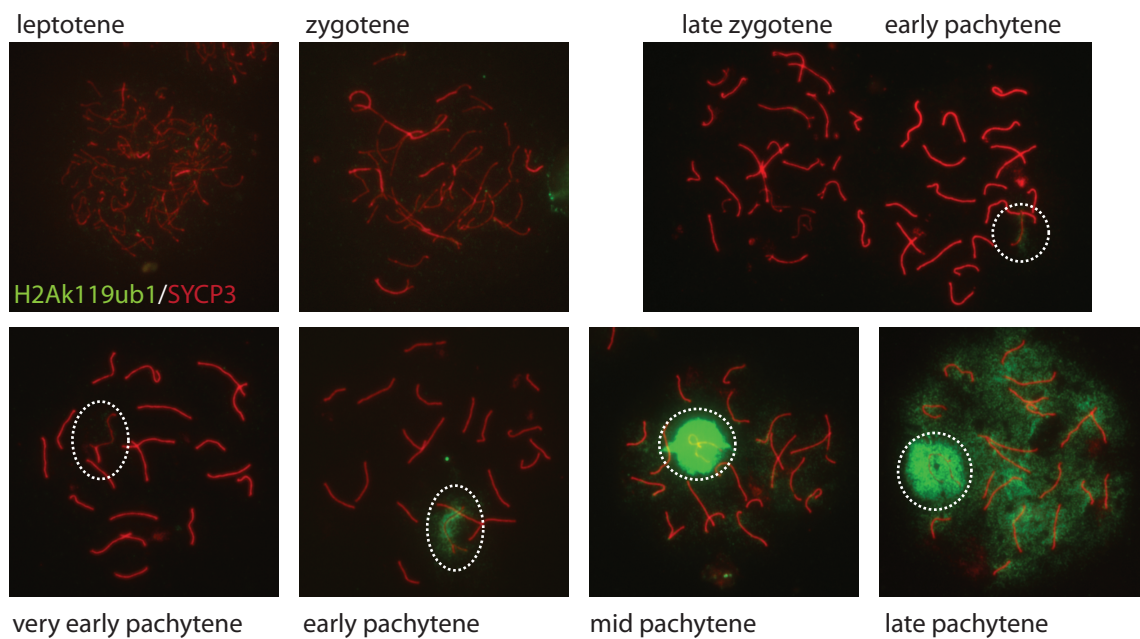

B

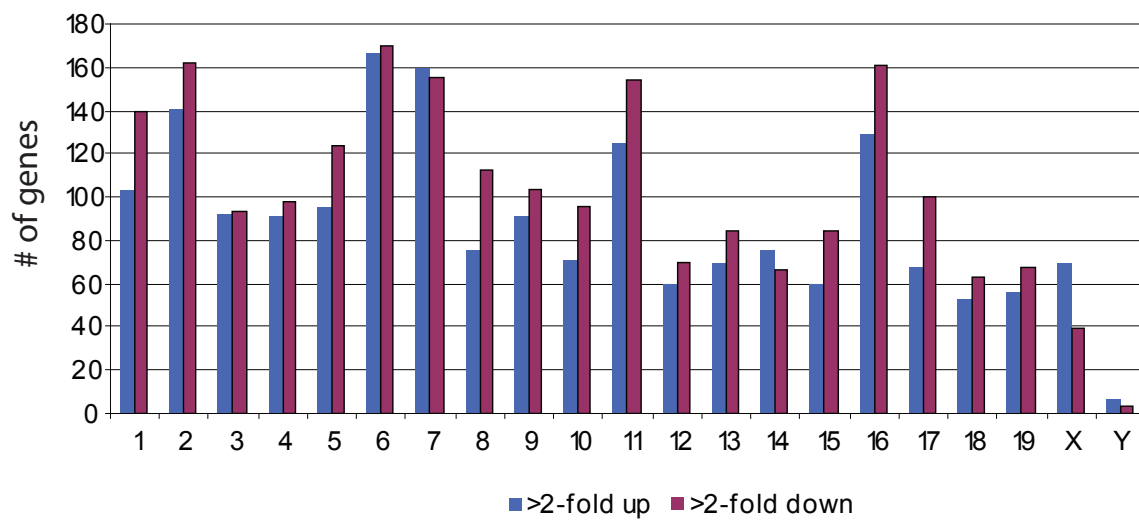

Supplement: Additional file 6 — Analyses of gene expression in Ubr2 knockout testis and H2AK119 ubiquitylation in wild type spermatocytes. A) Analysis of H2AK119 ubiquitylation (H2AK119ub1, green) and SYCP3 (red) during meiotic prophase in spread wild type mouse spermatocyte nuclei. Each image was obtained using the same microscope and camera settings. B) Genes that were more than two-fold up or downregulated in Ubr2 knockout versus wild type samples [44] were calculated per chromosome. [file 1471-2164-11-367-S6.PDF]
