# Supplementary material for: The ubiquitin-conjugating enzyme HR6B is required for maintenance of X chromosome silencing in mouse spermatocytes and spermatids
Source: BMC Genomics. 2010 Jun 10;11:367. doi: 10.1186/1471-2164-11-367 (PMC3091626; doi:10.1186/1471-2164-11-367)
Supplement: Additional file 7 — PCR primers used for qRTPCR. [file 1471-2164-11-367-S7.PDF]

Name	Forward primer	exon	Reversed primer	exon	Annealing temp (°C)	Product size
2610034M16Rik	AAGGAACTGGCGATTATCCC	6	TTCCTCAGCAGCACTATTGG	7	60	92
Cd24a	TTCTGGCACTGCTCCTACCC	2	CTGGTGGTAGCGTTACTTGG	2	60	112
AI987692/Gsdmc2	CCTGGATGAGCTGCGAAAGG	12	GCACCATCAGAGCTTGAAGG	12-13	60	86
Ripk4	GACTTTGAGGGCCGAACACC	8	GGCAACCAGGCATCCTTTCC	8	54	119
Tmcc2	GGAACTGCGGGAGATAAAGG	3	CAGGCACTGGGTCATGTAGG	3	54	103
Lzp-s/Lyz1	GCCTGTGGGATCAATTGC	2	CATGCTCGAATGCCTTGG	3	54	101
Gm614/Il2rg	AGACTCTGCCAGCAAATTCC	3	TTCAGCATGGGCACAATAGC	4	53	117
Rbmy1a1	GATCATGCTGGCTATCTTGG	9	TTGCAGATGGTGCCTCATGG	10	53	115
Sms	GCTGGACCTTCAGAGTTACG	3	CGAACTATGGGTGGTAATCG	4-5	54	138
4930408F14Rik	ACCTGGAGCTTGTGGATGG	1	CGAGGTACACTCTAGGTTTGG	1	54	117
Ssty	ATACCACAGCAAGGCTACAG	3	TCAGGGTGTTGGAAGAAGAC	3	58	107
Actin-β	CCGTGAAAAGATGACCCAG	3	TAGCCACGCTCGGTCAGG	4	54-61	249
Major satellite repeats	GACGACTTGAAAAATGACGAAATC		CATATTCCAGGTCCTTCAGTGTGC		59	308
Minor satellite repeats	CATGGAAAATGATAAAAACC		CATCTAATATGTTCTACAGTGTGG		52	162
Mariner	AGGCAGCAGAGCACAAATG		TTGCTGTTAAGGGAATTGTGG		61	198
Charlie	TTGAGAATCGGATGGGAGAC		AAGAACTGTCTTATTCAGGC		59	119
LTR transposon	AGCAGGTGAAGCCACTG		CTTGCCACACTTAGAGC		57	270
Line L1	TTTGGGACACAATGAAAGCA		CTGCCGTCTACTCCTCTTGG		57	155
rDNA	CCTGTGAATTCTCTGAACTC		CCTAAACTGCTGACAGGGTG		59	198
Sine B1	GTGGCGCACGCCTTTAATC		GACAGGGTTTCTCTGTGTAG		59	113
Sine B2	GAGATGGCTCAGTGGTTAAG		CTGTCTTCAGACACTCCAG		59	131

List of primers used for qRTPCR
